# Increasing prevalence of diabetes mellitus in a developing country and its related factors

**DOI:** 10.1371/journal.pone.0187670

**Published:** 2017-11-07

**Authors:** Worku Animaw, Yeshaneh Seyoum

**Affiliations:** School of Nursing, College of Medicine and Health Sciences, Bahir Dar University, Bahir Dar, Ethiopia; CUNY, UNITED STATES

## Abstract

**Background:**

All countries, irrespective of their developmental stage, face an increasing burden of non-communicable diseases including diabetes mellitus. There is substantial evidence of the existence of the gap in the level of diabetes mellitus and its complications prevention and control measures in developing countries. This study aimed to assess the prevalence of diabetes mellitus in urban and rural dwellers in a low-income country from both younger and older population and to identify factors related.

**Methods:**

This is a community based comparative cross-sectional study conducted in a low-income country, Ethiopia. The sample size was determined by EPI-Info for two populations; the WHO’s STEP-wise approach for non-communicable diseases surveillance in developing countries was employed for sampling, study variable selection and data collection procedures. Fasting blood glucose levels were measured by finger pricking after overnight fasting. Data entry was done by EPI-data computer program version 3.1 and then processed by SPSS version 20. Bivariate and multivariate logistic regression tests were used to assess the associations between diabetes status of individuals and its potential predictor variables. P-value < 0.05 was considered as statistically significant level.

**Result:**

The study was conducted on 1405 individuals with age range of 18–97 years old. The mean fasting blood glucose level for study participants was 91.16mg/dl; while it was 94.73mg/dl for urban and 87.71mg/dl for rural dwellers. The prevalence of diabetes mellitus was 3.3%; while it was 2.0% for rural and (4.6%) for urban dwellers. Both the mean blood glucose level and the prevalence of diabetes mellitus were significantly higher for urban residents than rural. More than three-fourths of diabetic cases were newly diagnosed by this study. Urban dwellers, centrally obese, overweight, and hypertensive individuals have higher odds of getting diabetes mellitus.

**Conclusions and recommendations:**

High prevalence of diabetes mellitus involving both old and young population was documented. Most diabetic cases were suddenly diagnosed during this survey. The problem is noticeably alarming, attention should be given to the control and prevention of diabetes mellitus and related complications.

## Introduction

Non-communicable diseases (NCDs) are becoming major health challenges with continually increasing burden [1]. Diabetes mellitus is one main segments of chronic non-communicable diseases [2]. In 2000, 60% of deaths and 43% burden of diseases in the world were due to NCDs [3]. Prediction made indicated that the burden of NCDs will increase about three-quarter of all deaths and 60% of all diseases globally by the year 2020 [4]. As reported by International Diabetes Federation (IDF), approximately 75–80% of people with diabetes die due to cardiovascular complications [5].

All countries, irrespective of their economic developmental, epidemiological and demographical variability, are facing an increasing burden of non-communicable diseases including diabetes mellitus [4]. Diabetes mellitus with other NCDs are responsible for an increasing burden of diseases in developing countries. In Sub-Saharan Africa, NCDs are predicted to exceed infectious diseases by the year 2030 [6]. It has been projected that the number of people with diabetes will increase to 300 million by 2025 and 366 million by 2030 from 171 million in 2000. The majority of these numerical increments will occur in developing countries [7–9]. Type 2 diabetes mellitus accounts for over 90% of diabetes and this proportion is higher if it is adjusted for older and urban population [10].

Studies around the world reported different level in the prevalence of diabetes mellitus. In Guatemala, the prevalence of diabetes was 8.4% where almost half of them (4.1%) were newly diagnosed [11]. In Bangladesh, a higher prevalence of diabetes was found among females, old age, centrally obese and urban dwellers [12]. A study conducted in Korea reported that 21.8% and 15.3% of participants had impaired fasting glucose level (IFG) and diabetes respectively [13]. The World Health Organization (WHO) estimated that the prevalence of diabetes in Kenya will rise from 3.3% in 2000 to 4.5% by 2025. The prevalence of diabetes mellitus type 2 in African countries ranged from 1% in rural Uganda [10] to 12% in urban Kenya. Screening studies found significant proportions (> 40%) of diabetic cases who were previously undiagnosed [10, 14]. WHO reported that there were about 800,000 people having diabetes in Ethiopia in 2000 and the number is expected to escalate to 1.8 million by the year 2030 [15].

A community based comparative study in Gondar found that the prevalence of diabetes mellitus among adults aged 35 years and above was 3.6%, while it was 5.1% for urban and 2.1% for rural dwellers. The majority (69%) of diabetic cases were newly diagnosed; with the highest proportion (82.6%) in rural residents [16].

Consumption of calorie-dense foods, sedentary lifestyle, tobacco consumption, older age, family history of diabetes and use of antiretroviral medications were the identified risk factors for metabolic syndrome in Gondar and Addis Ababa [16, 17]. Another study from southern Ethiopia found that hypertension, central obesity, and overweight had a strong association with diabetes mellitus [17].

The burden of diabetes and diabetes-related mortality and disability are rising in Africa. Sedentary lifestyles coupled with growing urbanization cultures and processed diets are predicted to triple the prevalence of diabetes mellitus in the coming 25 years involving young populations too [18, 19]. In Ethiopia, national data on prevalence and incidence of diabetes are lacking. However, patients attendances and admission rates due to diabetes mellitus are rising in hospitals. In the previous 2–3 decades, there have been observable lifestyle changes with significant population growth and urbanization which are the main risk factors repeatedly reported.

By the time diabetes-related complications become clinically manifested, it will be too late to overcome the complications; that also demands costly resources which is unaffordable in developing countries. Early detection, intervention and avoidance of risk factors have an enormous benefit which is only possible when there is evidence depicting the magnitude and risks of diabetes. However, most studies in Ethiopia were institution-based, focusing on urban dwellers and old age individuals only. Community-based epidemiological evidence incorporating urban and rural residents, younger and older population is essential to plan and intervene relying on evidence. This study aimed to complement this evidence gap in the study area. Hence, this study assessed the prevalence of diabetes mellitus in urban and rural dwellers and identified related factors.

## Methods and materials

Community based comparative cross-sectional study was conducted among individuals aged 18 years old and above in 2015. Pregnant, mother in post-partum period (6 weeks after delivery) and sick individuals during data collection period were excluded from the study. The sample size was determined by EPI-Info Statcalc for two population proportions. Taking the prevalence of DM from a study conducted in Gondar [16] 5.1% in urban and 2.1% in rural, considering 95% confidence interval, 80% power, one to one exposed to unexposed ratio and 10% of non-response rate; the total calculated sample size became 1472 (736 for urban and 736 for rural settings). WHO’s STEP-wise approach for non-communicable diseases (NCDs) surveillance in developing countries was employed for sampling approach [20]. According to the manual, a multistage sampling strategy was used to select study participants. In the first sampling stage (SS1) one rural and one urban districts were selected; in the second stage of sampling, 6 Kebels (smallest administrative unit) from each district were selected randomly; in the third stage, after proportionally distributing the required number of households (HHs) to each Kebele, HHs were systematically selected using “K” factor of eligible HHs. In the last stage of sampling (SS4), one eligible participant was selected from each of the selected HHs. This kind of sampling method is suggested by WHO’s STEP-wise manual [21]. Details of sampling procedures are presented in supporting file (S1 File).

Using adopted questionnaire data were collected by nurses after training. The questionnaire was adopted from WHO for non-communicable diseases (NCDs) surveillance in developing countries [20]. The adopted questionnaire was translated into Amharic language (local language) and pretested in 5% of calculated urban and rural sample sizes in similar setups. In accordance with the STEPs manual, questions related to alcohol and substance use were tailored and modified with few additional questions to reflect the local context of Ethiopia. After gaining written informed consent, data were collected in accordance with the STEP-wise approach; the approach has three levels: the first level is interview to gather sociodemographic and behavioral information, the second level is simple physical measurements (weight, height, waist circumference and hip circumference), and third is for the biochemical tests (blood glucose test) [20].

Each study participant was contacted for a minimum of two consecutive days. The first day was used for consent grant, interview, anthropometric/physical measurements and blood pressure measurement. In the first day contact, an appointment was made for the coming morning by instructing the participants not to take any food and fluid (fasting for a minimum of 8 hours) until blood sample was taken in the next morning contact.

Anthropometric measurements (height, waist and hip circumferences) were taken without heavy outdoor clothing. Stature was measured to the nearest millimeter using standard and caliber anthropometric rod. Weight was measured on a pre-standardized body weighing scale. The hip circumferences were measured at the maximum circumference around the hips and the waist circumferences were obtained at the level of the umbilicus at the midpoint between the lower margin of the last palpable rib and the top of the iliac crest (hip bone) using a measuring tape. Blood pressure (BP) was measured while participants sitting and resting for at least five minutes. Three BP measurements were taken with 3-5minutes interval between consecutive measurements and the average was taken for analysis.

The WHO recommends fasting blood glucose values for venous and capillary blood are identical, hence we used fasting capillary blood glucose test to determine participants’ diabetic status. In this study, as per the appointment made in the first contact day, each participant was re-visited in the second day for fasting blood glucose level measurement. Before taking the sample, we confirmed for the right participant and appropriate fasting status. Fasting blood glucose level was determined by finger pricking method using a “one-touch” glucose meter (SensoCard^®^) after an overnight fasting [20, 22]. If the first test was above the normal range, the test was repeated in the next day with the similar circumstance.

Data entered into EPI data version 3.1 and then transferred to SPSS; data cleaning, coding and analysis were performed using SPSS version 20 statistical software. Independent (blood glucose level) and dependent variables (blood pressure, BMI and Waist to Hip Ratio) were categorized using the definitions adopted from WHO [22]. Dependent variable (fasting capillary blood glucose level) was dichotomized into diabetic and non-diabetic while non-diabetic incorporated individuals with normoglycemic and impaired fasting glucose (IFG) level sharing the definition given by WHO. Individuals were considered as diabetic if the average of the two consecutive fasting blood glucose level was above 126 mg/dl; otherwise considered as non-diabetic [22]. Detail operational definitions for variables used in the study are attached in supplemental materials. To explain the study population in relation to relevant variables, frequencies and summary statistics were used. Associations between the diabetic status of individuals and its potential predictor variables were assessed and presented using logistic regression tests. From bivariate logistics regression test model, only variables showed significant correlation/association with dependent variable were entered into the final multivariate regression model. P-value below of 0.05 was considered as the statistically significant cut-off point.

## Results

In this study, a total 1472 adult participants were recruited and 1405 of them have fully participated in the study which gives 95.5% response rate. As it is presented in Table 1, half of the participants were urban dwellers. The median age of the participants was 33 years old ranging from 18–97 years old. Age of the participants was categorized using four percentiles. Though the categorization system is different, more than forty percent of the current study participants were below the age of thirty which is close to the national and regional age distributions [23, 24]. Seven hundred and ninety-six (56.7%) of all participants were female. In the study area male to female ratio was reported to be about 1 to 0.8 in the previous reports [23, 24] which is close to the sample of this study (1 to 0.76). Two-thirds of the participants were married which is similar to the regional percentage [23], 30.2% of participants were farmers by their occupation.

**Table 1:** Sociodemographic characteristics of study participants (N = 1405).

		Frequency	Valid percent
Residence	Urban	691	49.2
	Rural	714	50.8
Sex	Male	609	43.3
	Female	796	56.7
Age	18–23	297	21.1
	24–28	271	19.3
	29–36	254	18.1
	37–50	312	22.2
	>50	271	19.3
Marital status	Single	321	22.9
	Married	924	65.8
	Divorced	72	5.1
	Widowed	84	6.0
	Other	3	0.2
Academic status	Unable to read and write	525	37.4
	Read and write only	208	14.8
	Grade 1–8	244	17.4
	Grade 9–12	227	16.2
	Diploma and above	201	14.3
Religion	Orthodox	1287	91.6
	Muslim	98	7.0
	Protestant	19	1.4
	Other	1	0.1
Occupation	Student	112	8.0
	Employed	161	11.5
	Merchant	142	10.1
	Farmer	424	30.2
	House wife	349	24.8
	Daily laborer and have no regular occupation	217	15.4

The participants’ substance use status was also assessed; the majority of participants (81.1%) drank alcohol, 5.1% chewed Khat, and 13 (0.9%) smoked cigarette at least once in their life. The mean (±SD) weight of participants was 55.0 kg (± 9.08) with minimum and maximum of 32.8 and 97.8 kg, respectively. The mean, maximum and minimum height of participants was 1.6, 1.27 and 1.93 meter respectively. The mean (±SD) of Body Mass Index (BMI) of participants was 21.1Kg/m^2^ (±3.24) with minimum and maximum of 10.3 and 35.7kg/m^2^ respectively. The BMI status of the participants was categorized into four categories from underweight to obese, as defined in attachment (Operational definitions in S1 File). Accordingly, 67.7% of the participants had normal weight, while 20.8%, 9.3% and 2.1% were underweight, overweight and obese, respectively. As depicted in Table 2, 61% of the participants had normal range of waist to hip ratio. Nearly half (48.8%) of participants were identified as pre-hypertensive (37.4%) and hypertensive (11.4%). The mean fasting blood glucose level for the study participants was 91.16mg/dl with the standard deviations of ±21.38, while the maximum and minimum levels were 350mg/dl and 30mg/dl, respectively.

**Table 2:** Anthropometric and other body measurement results of participants (N = 1405).

		Frequency	Valid percent
Body Mass Index (BMI)	Normal weight	950	67.7
	Under weight	293	20.8
	Over weight	130	9.3
	Obese	30	2.1
Waist to hip ratio (WTHR) status of both sex	Normal WTHR	857	61.0
	Centrally obese	547	38.9
	Missing	1	0.1
Blood pressure	Normotensive	719	51.2
	Pre-Hypertensive	526	37.4
	Hypertensive	160	11.4
Blood glucose level	Normoglycemia	1311	93.3
	Impaired Fasting Glucose (IFG)	48	3.4
	Diabetes Mellitus (DM)	46	3.3

**Fig 1:**
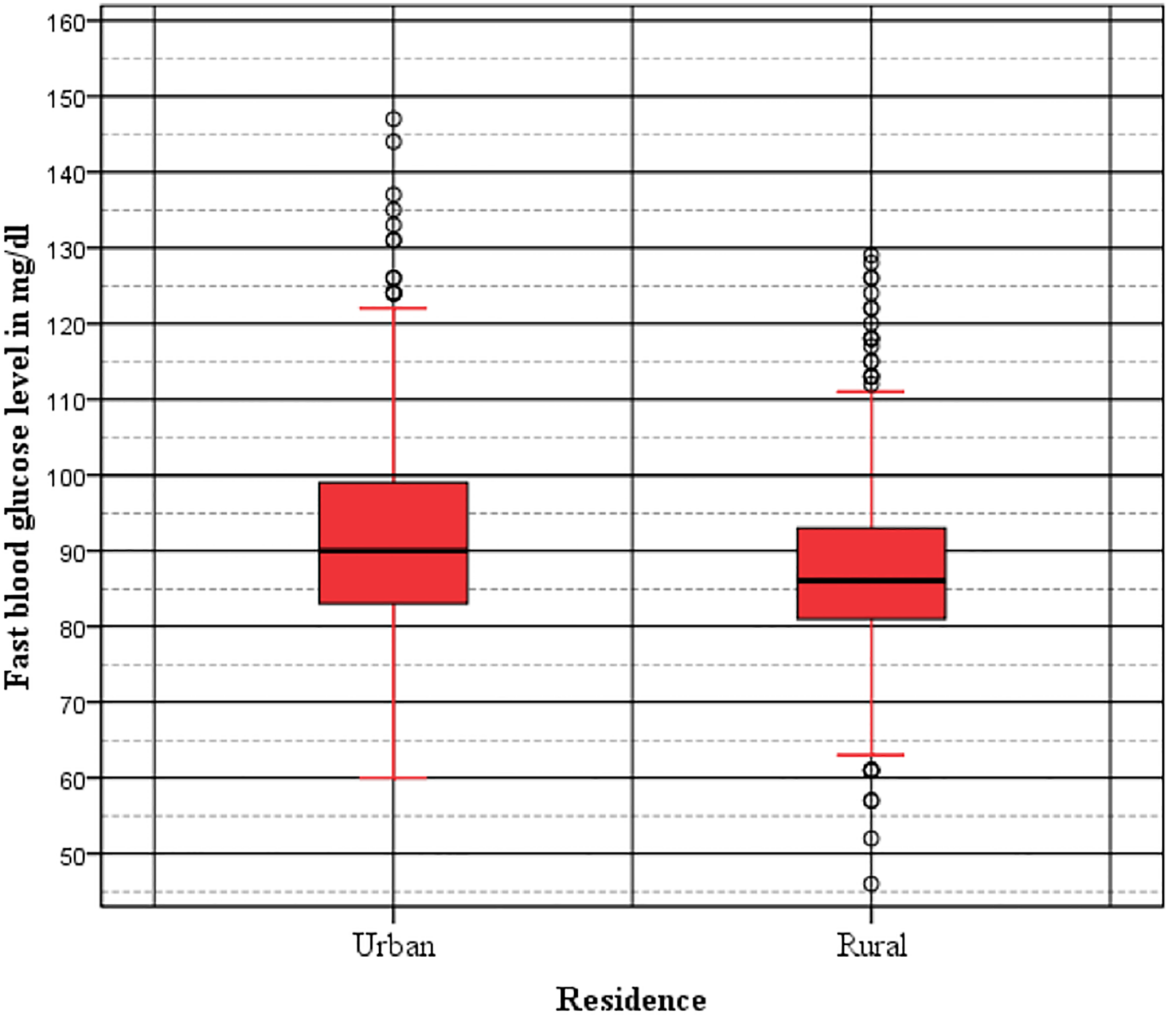
Boxplot of fasting blood glucose level for urban and rural dwellers.

The mean fasting blood glucose level of urban residents was 94.73mg/dl with the first and third quartile of 82.00mg/dl and 99.00mg/dl respectively. While the mean fasting blood glucose level for rural dwellers was 87.71mg/dl with first and third quartile of 81.00mg/dl and 93.00 mg/dl respectively. As depicted in boxplot of Fig 1 there was a significant mean difference (p-value < 0.001) between urban and rural dwellers. While drawing the boxplot extreme outliers in blood glucose level were excluded. As shown in the boxplot (Fig 1) lower outliers were only from the rural residents. The upper outliers were from both residency sites. However, the outliers from the rural participants were more concentrated and all below 130 mg/dl; while most outliers for the urban residents were above this level and more scattered indicating abnormally high blood glucose level among the urban residents.

Mean fasting blood glucose level for male participants was 90.70 mg/dl with standard deviations of ± 21.52 while it was 91.52 mg/dl (± 21.21) for females. The prevalence of diabetes mellitus was 3.4% for males and 3.1% for females but the difference was not statistically significant.

Only 104 (7.4%) among all the study participants had ever tested for their blood glucose level. Of 46 identified diabetic cases only 16 (34.8%) had previous test for their blood glucose level, the rest 30 never had blood glucose test. More than three-fourths (76.1%) of participants were newly identified diabetic cases; only 11(23.9%) of the identified diabetic cases knew they had diabetes mellitus before the test on this study and almost all (10/11) of them were urban dwellers.

When fasting blood glucose levels were categorized as defined in the supplemental attachment, it was found that 46(3.3%) and 48(3.4%) of 1405 participants were diabetic and pre-diabetic (impaired fasting glucose level) respectively. To identify factors related to diabetes mellitus, first bivariate logistic regression tests were computed and presented in Table 3. In these tests sex, occupation, vigorousness of daily activity, academic level and Khat consumption status of participants did not show a statistically significant difference and hence these variables were excluded from final regression model (multiple logistic regression) tests.

**Table 3:** Diabetes mellitus and its’ association (N = 1405).

		Dichotomized DM status		OR (95% CI)	
		None DM N (%)	DM N (%)	Crude	Adjuster
Residence	Urban	659(95.4)	32(4.6)	1	1
	Rural	700 (98.0)	14 (2.0)	0.41(0.22, 0.78)	0.43(0.21, 0.87)
Sex	Male	588 (96.6)	21(3.4)	1	1
	Female	771(96.9)	25(3.1)	.91(0.50, 1.63)	
Age	[18–23]	293(98.7)	4(1.3)	1	1
	[24–28]	266(98.2)	5(1.8)	1.37(0.36, 5.18)	1.49(0.38, 5.81)
	[29–36]	252(99.2)	2(0.8)	.58(0.11, 3.20)	0.68(0.11, 4.16)
	[37–50]	294(94.2)	18(5.8)	4.48(1.50, 13.41)	5.55(1.57, 19.66)
	>50	254(93.7)	17(6.3)	4.90(1.62, 14.75)	6.34(1.14, 19.78)
Marital status	Single	318(98.1)	6(1.9)	1	1
	Married	893(96.6)	31(3.4)	1.84(0.76, 4.45)	1.03(0.35, 3.01)
	Divorced	69(95.8)	3(4.2)	2.30(0.56, 9.44)	1.09(0.23, 5.16)
	Widowed	78(92.9)	6(7.1)	4.07(1.28, 12.98)	1.11(0.27, 4.45)
Academic status	Illiterate	508 (96.8)	17(3.2)	1	
	Read and write only	201(96.6)	7(3.4)	1.04(0.42, 2.54)	
	Grade 1–8	235(96.3)	9(3.7)	1.14(0.50, 2.60)	
	Grade 9–12	222(97.8)	5(2.2)	.67(0.24, 1.85)	
	Diploma and above	193(96.0)	8(4.0)	1.24(0.53, 2.98)	
Occupation	Employed	153(95.0)	8(5.0)	1	
	Student	111(99.1)	1(0.9)	.17(0.02, 1.390	
	Merchant	135(95.1)	7(4.9)	.99(0.35, 2.81)	
	Farmer	417(98.3)	7(1.7)	0.32(0.11, 1.01)	
	House wife	337(96.6)	12(3.4)	.68(0.27, 1.70)	
	No occupation	206(94.9)	11(5.1)	1.02(0.40, 2.60)	
Vigorous activity	Yes	730(97.6)	18(2.4)	1	
	No	629(95.7)	28(4.3)	1.80(0.99, 3.29)	
Ever drink alcohol	Yes	1109 (97.4)	30(2.6)	1	1
	No	250(94.0)	16(6.0)	2.36(1.27, 4.41)	1.81(0.92, 3.57)
Ever chewed Kchat	Yes	67(93.1)	5(6.9)		
	No	1291 (96.9)	41(3.1)	.42(0.16, 1.11)	
Body Mass Index (BMI)	Normal weight	927(97.6)	23(2.4)		
	Under weight	285(97.3)	8(2.7)	1.12(0.49, 2.54)	1.26(0.55, 2.87)
	Over weight	117(90.0)	13(10.0)	4.47(2.20, 9.8)	2.89(1.36, 6.11)
	Obese	28(93.3)	2(6.7)	2.88(0.65, 12.81)	1.50(0.32, 7.08)
Central obesity	Normal	839(97.9)	18(2.1)		
	Central obese	519(94.9)	28(5.1)	2.51(1.37, 4.59)	1.86(0.98, 3.52)
BP status	Normotensive	704(97.9)	15(2.1)		
	Pre-Hypertensive	511(97.1)	15(2.9)	1.38(0.66, 2.84)	1.15(0,55, 2.41)
	Hypertensive	144(90.0)	16(10.0)	5.21(2.51, 10.78)	3.55(1.63, 7.70)

Though the association test did not show a statistically significant difference, only 2.4% of participants whose work involved vigorous activity were identified to be diabetic, while it was nearly double (4.3%) for those participants whose work did not involve vigorous activity. Six (1.6%) of the single participants by their marital status were found to be diabetic as compared to 3.4%, 4.2% and 7.1% of married, divorced and widowed participants respectively. Though the first association test showed statistically significant difference among individuals with different marital statuses, it could not maintain its significant difference while it was adjusted with other variables.

The prevalence of diabetes mellitus was 2.0% for the rural dwellers, while it was more than double (4.6%) for urban residents. This difference was also statistically significant with adjusted odds ratio (AOR (95% CI)) of 0.043(0.21, 0.87). The prevalence of diabetes was different among age categories, ranging from 0.8% in the age category 29–36 years old to 6.3% among participants aged 50 years and above. Multiple logistic regression tests also revealed that the statistically significant difference among age categories. Participants aged 37 to 50 years old were found about six-fold odds to be diabetic AOR (95% CI) is 5.5(1.5–19.6) as compared with participants aged 18–23 years old. The odds of being diabetes continues to increase to more than six folds when participants get older than 50 years.

Body weight was among the known risk factors frequently identified by different researchers. The current study also confirmed that individuals’ weight was one of the associated factors in current study populations too. Only 2.4% of normal weight participants were found to be diabetic while it was 10.0% for overweight individuals. The multiple logistic regression tests also revealed that there was a significant difference among weight-to-height (BMI) categories of participants. Overweight individuals were nearly three times more likely to have DM than normal weight individuals AOR (95%C) = 2.89(1.36, 6.11). As overweight and obese, centrally obese individuals were also at great risk of many systemic and metabolic disorders. In this study, a higher prevalence of diabetes mellitus (5.1%) was documented among centrally obese individuals, which is more than double of individuals who were not centrally obese.

## Discussions

Data from 1405 adults were collected in accordance with the STEP-wise approach as recommended by the World Health Organization (WHO) for non-communicable diseases (NCD) surveillance in developing countries [20]. The representativeness of the sample was assured by proper sampling strategies and comparing important sociodemographic characteristics of the sampled participants such as gender, age, and marital status with the region and countrywide populations.

This study disclosed high prevalence of DM in the study area and population. The prevalence of DM in the current study population (18 years old and above) was 3.3%. This percentage is nearly equivalent with other previous studies conducted in other countries; Guatemala [11], Kenya [10], and other societies of Ethiopia [16]. However, the previous studies involved only adult population aged 36 years and above in contrast to the current study which involved younger participants too. In the current study, the percentage of DM among individuals 36 years and above was 6.0% which is almost double of other previous studies and significantly higher when compared with national estimations of DM which is 1.9% [15]. This indicated an increment in the magnitude of DM over time and to the younger population.

Prevalence of DM was repeatedly reported as significantly different among urban and rural dwellers in the previous studies. The current study also revealed that the difference among rural and urban dwellers was significant; 2.0% of rural and 4.6% of urban dwellers had DM. The difference is in line with other previous studies [10, 14, 16] and statistically significant. Nonetheless, this magnitude is inclusive to young population, unlike other previous studies which were done only in adults older than 36 years.

Another important finding was that more than three fourth (76.1%) of the DM cases were newly diagnosed by this study which is higher than the previous study [16]. All diabetic cases identified (except 1) from rural area were first diagnosed by this survey. This is very alarming for anyone concerned. If this survey was not conducted and if these participants were not included in the sample/survey, it means that these diabetic cases would seek health care after complications start to occur. That time would be too late to reverse diabetic-related complications.

Alcohol consumption was one of the factors identified by earlier studies to be related to the diabetic status of individuals. The current study also disclosed that 2.6% of participants ever consumed alcohol in their lifetime were found to be diabetic, while the percentage of diabetic cases among participants never drank alcohol were near to three-fold of it (6.2%). The first logistic association test showed alcohol consumers seemed to have less chance to get diabetes mellitus. This controversial finding was also reported in a study conducted a year before current study in the community with similar socio-demographic characteristics [16]. However, the relationship between alcohol consumption and diabetic status was not maintained while it was adjusted with other variables AOR (95% CI) = 1.81(0.92, 3.57).

Systemic diseases are repeatedly reported to occur concomitantly. These diseases also have many shared risk factors and even one disease causes for the occurrence of the others. Being overweight and centrally obese were significantly related with diabetes status of individuals which was in line with other previous studies [12, 16]. Higher blood pressure status and blood glucose level are among pillars of systemic diseases known to happen together. This study also ratifies that these two problems occurred together. Hypertensive individuals had 3.55 times more likely to be diabetic also AOR (95%CI) = 3.55(1.65, 7.70) this is in-line with the previous study in the Southern Ethiopia [17]. This study revealed these fatal diseases are occurring concomitantly, albeit as limitations of a cross-sectional survey, this study could not assure which disease occurred first.

This study was conducted in a large number of participants with proper sampling strategies which could enhance the representation of the study population. WHO’s recommendation for fasting blood glucose and anthropometric measurements have been strictly followed. Standardized measuring tools were used. Involving younger populations from both urban and rural area could demonstrate the distribution of the problem to the general population. As a cross-sectional survey, this study could not test cause and effect relationships of the diabetic status and its related factors. Another limitation of this study may be that other factors such as malnutrition at childhood and viral infection (like HIV) which may be associated with diabetes mellitus but were not recorded in this study. Activity and alcohol and substance consumption practices were only assessed by interview which may not be accurate due to recall bias.

## Conclusions and recommendations

This study disclosed high prevalence of diabetes mellitus (DM) in the study area and population with higher prevalence in the urban population. An increment in the magnitude of DM over time and to younger population is documented; indeed, as age increase odds of having DM increases in about 6-fold. Urban dwellers, centrally obese, overweight, and hypertensive individuals have higher odds of getting diabetes mellitus. Community, particularly rural community are not having the test for their glucose level until it gets complicated. Most of the DM cases identified in this study were newly diagnosed. Blood glucose level test practice is very poor in the study area and we suggest the screening practice shall be promoted in order to prevent diabetes-related complications. Further study is recommended to identify what is special about the urban dwellers to have a higher risk of DM other than activity and feeding habit.

## Ethics approval and consent to participate

Ethical approval was obtained from Institutional Review Board of Bahir Dar University prior to enrolment. Written consent was sought from each participant. Every identified diabetic and pre-diabetic and/or hypertensive and pre-hypertensive individuals were advised to visit health institution as soon as possible and we arranged conditions to visit health institution when necessary. Data are kept confidential and communicated without disclosing individual identity.

## Supporting information

S1 File Schematic presentation of sampling techniques. (DOCX)
