# Supplementary material for: Increasing prevalence of diabetes mellitus in a developing country and its related factors
Source: PLoS One. 2017 Nov 7;12(11):e0187670. doi: 10.1371/journal.pone.0187670 (PMC5675402; doi:10.1371/journal.pone.0187670)
Supplement: S1 File — (DOCX) [file pone.0187670.s001.docx]

**Operational Definitions**

**Normoglycemia:** capillary fasting blood glucose level < 110mg/dl

**Impaired Fasting Glucose (IFG**): average of two consecutive capillary fasting blood glucose level [110–125mg/dl]

**Diabetes Mellitus:**  average of two consecutive fasting blood glucose level (FPG)> 126 mg/dl

**Underweight:** BMI<18.5 kg/m^2^

**Normal Weight**: BMI 18.5–24.9 kg/m^2^

**Overweight**: BMI 25.0–29.9 kg/m^2^

**Obese**: BMI >30.0).

**Centrally obese**: waist-hip ratio (WHR) > 0.95 for men and 0.85 for women

**Normotensive***: systolic BP<120mmHg and diastolic BP<80mmHg)

**Pre-hypertension***: average of two consecutive systolic BP 120–139mmHg or diastolic BP 80–89mmHg);

**Hypertension***: average of two consecutive systolic BP ≥140 mmHg or diastolic BP ≥90mmHg)

***** *if test result falls in different categories either for systolic or diastolic BP measurement the highest measurement value will be used to categorise individuals BP status.*

**Figure A. Sampling procedure**

Target Population (18 years and above) (N=1472)

SS1

1 Rural District

1 Urban District

SS2

| 6 Kebeles (N=736) |
| --- |

6 Kebeles (N=736)

| Ka  115hhs | Kb  125hhs | Kc  125hhs | Kd  125hhs | Ke  126hhs | Kf  120hhs |
| --- | --- | --- | --- | --- | --- |

| K1  130hhs | K2  125hhs | K3  121hhs | K4  130hhs | K5  150hhs | K6  115hhs |
| --- | --- | --- | --- | --- | --- |

SS3

| 1 study unit/individual from each hhs (N=736) |
| --- |

| 1 study unit/individual from each hhs (N=736) |
| --- |

SS4

- K: Kebeles (Smallest administrative unite in the country (Ethiopia)
- hhs: households
- SS: Sampling stage

S1 Figure: Schematic presentation of sampling techniques
